# Untargeted Lipidomic Profiling of Amniotic Fluid Reveals Dysregulated Lipid Metabolism in Healthy Normal-Weight Mothers with Fetal Macrosomia

**DOI:** 10.3390/nu16223804

**Published:** 2024-11-06

**Authors:** Isra’a Haj-Husein, Stan Kubow, Kristine G. Koski

**Affiliations:** School of Human Nutrition, McGill University, Ste-Anne de Bellevue, QC H9X 3V9, Canada; stan.kubow@mcgill.ca (S.K.); krsitine.koski@mcgill.ca (K.G.K.)

**Keywords:** lipidomics, lipids, fetal macrosomia, birth weight, amniotic fluid

## Abstract

Background: Alterations in maternal lipid metabolism have been elucidated by several studies in relation to macrosomia. However, the lipidome of the intrauterine compartment associated with macrosomia, particularly in early pregnancy, remains largely unknown. Objectives: (1) To compare the lipidomic profile of early 2nd trimester amniotic fluid (AF) of healthy mothers with normal body mass index who gave birth to large-for-gestational age (LGA) versus appropriate-for-gestational age (AGA) infants; and (2) to examine if insulin and glucose concentrations in AF were associated with the AF lipidomic profile. Methods: In this nested case–control study, bio-banked AF samples were collected from pregnant women undergoing routine amniocentesis at 12–22 weeks of gestation. A subsample of 15 LGA infants (cases) were contrasted with 15 AGA infants (controls). An untargeted lipidomics analysis using liquid chromatography quadrupole time-of-flight mass spectrometry was conducted. Univariate and multivariate statistical analyses (principal component analysis and partial least-squares discriminant analysis) were used to extract differentially abundant (DA) features with high variable importance in projection (VIP) scores. Results: LGA AF was characterized by elevations of 30 phosphatidic acid species. Among other DA features, sphingomyelin (SM 14:0;O2/20:1) had the highest VIP score and was markedly elevated in LGA AF. Neither insulin nor glucose was associated with 2nd trimester AF lipidomic profiles in these healthy, normal-weight mothers. Conclusion: These findings provide evidence of early dysregulated lipid metabolism in healthy, normal-weight mothers with LGA infants.

## 1. Introduction

Macrosomia and large-for-gestational age (LGA) are alternative terms used to describe fetal overgrowth, with higher risk for cesarean section, fetal shoulder dystocia [1], obesity, type 2 diabetes mellitus, and cardiovascular diseases later in life [2,3]. While maternal obesity and diabetes are strong factors, epidemiological studies emphasize a wide spectrum of maternal characteristics in association with macrosomia. In fact, a large proportion of LGA infants are born to mothers with normal glucose tolerance and body mass index (BMI) [4,5]. Macrosomia has often been viewed as a physiological response to maternal hyperglycemia, although it does not fully explain it even in diabetic pregnancies [6]. The role of maternal lipid metabolism, on the other hand, has become well recognized, with evidence indicating that its markers predict birth weight and fetal adiposity better than glucose [7,8]. Mothers of macrosomic infants were also found to have higher placental gene and protein expression levels of fatty acid binding and transport proteins, which indicate upregulated placental lipid transport and metabolism [9].

Several lipidomics studies have revealed significant alterations in maternal blood lipids in direct relation to fetal overgrowth [10,11]. For instance, reductions in different species of phosphatidylcholines, lysophosphatidylcholines, and lysophosphatidylinositols have been observed in early pregnant mothers in association with high birth weights [12,13,14]. These changes align with the lipidomic features associated with insulin resistance [15,16,17]. Alterations during the second half of pregnancy are complex and not consistent, but overall, they also indicate insulin resistance. Maternal short-chain acylcarnitines, such as acetylcarnitine, were reported to positively correlate with birth weight [18,19]. It was suggested that increased muscular fatty acid oxidation resulting from insulin resistance accumulates acetyl-CoA, which may justify the observed increase in acetylcarnitine [20]. Nevertheless, researchers investigated either the maternal side or the intrauterine compartment (i.e., cord blood/placenta) specifically at birth. The lipidome of the intrauterine compartment characterizing macrosomia at early pregnancy is still unknown and has the potential to provide further insights into its pathophysiology.

Amniotic fluid (AF) is thought to arise as a transudate of maternal and/or fetal plasma [21]. It uniquely reflects the interaction between maternal, placental, and fetal metabolism [22], especially at early pregnancy before fetal skin keratinization is fully attained at 25 weeks of gestation, which ends the bidirectional flow of fluids across the skin [23]. Moreover, fetal swallowing of AF, which begins as early as 16 weeks [23], was found to impact fetal growth, and conditions of esophageal ligation or atresia of the gastrointestinal tract are associated with lower birth weights [24,25]. Such dynamically changing fluid offers the potential to elucidate the metabolic perturbations affecting the intrauterine compartment in adverse maternal and fetal health conditions. Although it is considered an underutilized resource, given the invasive nature of amniocentesis as a procedure [26], several studies have provided important metabolic clues about gestational diabetes, prematurity, and other conditions using AF [27,28]. In the context of gestational diabetes, for example, profiling the AF metabolome provided evidence for the divergence between maternal and intrauterine lipid metabolism, with elevations in AF saturated and monounsaturated sphingomyelins (SMs) [27], whereas similar SM species were reported as lower in maternal circulation [20,29]. Such findings highlight the complexity of the associated metabolic dysregulation.

To date, there are no studies profiling the human AF lipidome in relation to macrosomia. Therefore, our objective was to compare the lipidomic profile of AF obtained at early second trimester between healthy normal-weight mothers who gave birth to LGA and AGA infants. At the same time, we aimed to examine if AF insulin and glucose concentrations were associated with these AF lipidomic profiles.

## 2. Materials and Methods

### 2.1. Study Design

In this nested case–control study, AF samples were selected from a biobank of samples previously collected from pregnant women who had undergone routine amniocentesis at early second trimester. Our inclusion criteria included healthy mothers with normal BMI (18.5–24.9 kg/m^2^) [30] who gave birth to either LGA or AGA infants. Women who had pre-existing health conditions, such as pre-pregnancy diabetes or other endocrine disorders, and those who developed gestational diabetes or hypertensive disorders of pregnancy were excluded. This exclusion criterion aimed to eliminate the confounding effect of these disorders and to better isolate the pathological mechanisms mediating LGA development. All relevant information was extracted from the participants’ medical records. A sample of 15 LGA infants (cases, birth weight >90th percentile for gestational age and gender) was randomly selected and matched with 15 AGA infants (controls, birth weight between 10th and 90th percentile for gestational age and gender). This sample size was deemed an acceptable starting point for our study given the exploratory nature of our work [31]. Individual 1:1 matching was conducted based on sex, amniocenteses week or the gestational age at the time of amniocenteses (±1 week), pre-pregnancy maternal BMI (±2 kg/m^2^), and maternal age (±2 years). A flowchart illustrating sample selection from the AF biobank is provided in Supplementary data (Figure S1). Samples were shipped in dry ice for lipidomic profiling at The Metabolomics Innovation Center (TMIC, Edmonton, AB, Canada).

### 2.2. Study Population and Characteristics of the AF Biobank

Participants’ recruitment was carried out in 2000–2004 at St. Mary’s Hospital Centre (Montreal, QC, Canada). Ethics approval was obtained from the McGill University and St. Mary’s Hospital Centre Institutional Review Boards. Pregnant women were recruited while undergoing routine amniocentesis for age-related genetic testing (15.1 ± 0.1 weeks, range 12–22 weeks). Women with multiple pregnancies and fetuses with genetic anomalies were exc1uded. Consents were obtained from participating women to allow the investigators to analyze their AF for various constituents on the remaining fluid once genetic testing was completed. The consent form also provided permission to obtain information from medical charts on maternal health, age, height, and pre-pregnancy weight, and infant sex, birth weight, and gestational age at delivery. As birth weight can be influenced by gestational length and fetal sex, Kramer birth weight percentile charts were used to control for these factors [32]. Aliquots of AF samples were stored at −80 °C since collection. Samples with meconium staining or blood were excluded. AF samples were analyzed for glucose and insulin as previously described [33]. Briefly, glucose was analyzed using an adapted hexokinase assay kit (No. 6082; Abbott Laboratories, North Chicago, IL, USA) for use with a microplate reader. Insulin was analyzed using the Beckman Access Ultrasensitive assay system (Beckman Coulter, Brea, CA, USA), which measures insulin to within 0.03–300 µIU/L.

### 2.3. Global Lipidomics Analysis

For lipid extraction, a modified Folch liquid–liquid extraction protocol was applied [34]. Each aliquot of 200 μL of sample was evaporated to dryness before its weight was determined. A corresponding amount of water was added to samples to resuspend before mixing with dichloromethane, methanol, and an internal standard mixture, which consisted of 15 deuterated lipids from different lipid subclasses: lysophosphatidylcholine (LPC d5-18:1), lysophosphatidylethanolamine (LPE d5-18:1), fatty acid (FA d3-16:0), cholesterol-d3, phosphatidylglycerol (PG d5-16:0/18:1), phosphatidylserine (PS d5-16:0/18:1), phosphatidic acid (PA d5-16:0/18:1), phosphatidylinositol (PI d5-16:0/18:1), ceramide (Cer d3-16:0/18:1), phosphatidylcholine (PC d5-16:0/18:1), phosphatidylethanolamine (PE d5-16:0/18:1), monoglyceride (MG d5-18:1), diglyceride (DG d5-18:1/16:0), triglyceride (TG d5-16:0/18:1/16:0), steryl ester (CE d3-18:1). A clean-up step was performed with water. Samples were equilibrated at room temperature for 10 min and centrifuged at 16,000× *g* for 10 min at 4 °C. An aliquot of the organic layer was evaporated to dryness with a nitrogen blowdown evaporator. The residue was immediately re-suspended in mobile phase B (10 mM ammonium formate in 95:5 (*v*/*v*) isopropanol:water), vortexed for 1 min, and diluted with mobile phase A (10 mM ammonium formate in 5:4:1 (*v*/*v*) methanol:acetonitrile:water).

Reversed-phase chromatography was conducted using ultra-high-performance liquid chromatography (UHPLC) with a Vanquish Flex Duo Tandem Binary UHPLC system (Thermo Fisher Scientific, Waltham, MA, USA) and a Waters Acquity CSH C18 column (5 cm × 2.1 mm, 1.7 μm particle size). Microflow-based lipidomic profiling of extracts was performed using UHPLC linked to a Bruker Impact II Quadrupole time-of-flight (QToF) mass spectrometer (Bruker Daltonics, Billerica, MA, USA), with separate injections for each ionization polarity and the following conditions: injection volume of 4.0 μL for positive ionization and 12.0 μL for negative ionization; flow rate of 210 to 300 μL/min; NovaMT 20-min-gradient; column temperature of 45 °C; and a mass range of 150–1500 *m*/*z*. MS/MS analysis was performed in auto-MS/MS mode with collision energies of 10–60 eV. Chromatograms were processed with an intensity threshold of 3000 cts; signal-to-noise ratio threshold of 3; minimum peak length of 6 spectra; retention time tolerance of 6 s for correction and 4 s for alignment; *m*/*z* tolerance of 20.0 ppm for peak picking and 20.0 ppm and 5.0 mDa for alignment. MS/MS spectra were acquired for all samples for alignment and identification using NovaMT LipidScreener V1 (Nova Medical Testing Inc., Edmonton, AB, Canada) [35].

For quality control (QC), a pooled mixture composed of one aliquot from each sample was prepared. The pooled mixture was split into multiple aliquots of equal volume, evaporated to dryness with a nitrogen blowdown evaporator, purged with nitrogen, and stored at −80 °C. One QC aliquot was extracted with each randomized batch of samples; 13 samples per batch, as this analysis was part of a larger project with a larger cohort of samples. Samples within each batch were injected in-between two injection replicates of the corresponding QC aliquot. Multiple QC aliquots were also injected before and after all samples to ensure technical stability.

### 2.4. Data Processing

The detected features from all samples were merged into one feature-intensity table. A three-tier annotation approach based on MS/MS spectral similarity match, retention time, and accurate mass match was employed for lipid identification. A filtering and scoring approach embedded in NovaMT LipidScreener was employed to calculate MS/MS match scores (when available), restrict the number of matches, and select the best identification. The approach uses parameters like the expected retention time range, expected adducts and isoforms, fatty acyl chain length, ionization efficiency and sensitivity of each lipid subclass, and *m*/*z* error to make the best identification choice for each feature among all isomeric and isobaric possibilities [35].

Features identified in Tier 1 and 2 were based on MS/MS identification. Specifically, Tier 1 included features that had an MS/MS match score of ≥500 and a precursor *m*/*z* error ≤ 20.0 ppm and 5.0 mDa. Features with an MS/MS match score of <500 and a precursor *m*/*z* error ≤ 20.0 ppm and 5.0 mDa were included in Tier 2. Features not identified by MS/MS (Tier 3) were imported into the Lipid Maps database for identification based on accurate mass match and a *m*/*z* error of ≤ 20.0 ppm and 5.0 mDa. All features identified in Tiers 1, 2, and 3 were combined for normalization and statistical analysis. The classification and shorthand notation of lipids followed the guidance of LipidMaps, MSDial and the Update on LIPID MAPS classification, nomenclature, and shorthand notation for MS-derived lipid structures [36,37].

### 2.5. Data Analysis

Missing values were substituted by (1) the median intensity of the sample group (LGA/AGA) for features detected in at least 75% of injections within the group; (2) the minimum intensity within the group for features detected in at least 50% of injections; or (3) the global minimum for all samples and QCs for features detected in less than 50% of injections within the group. Features not detected in ≥80% of injections within at least one sample group or QCs were filtered out.

To correct for ion suppression effects and any sources of technical variations, the identified features/lipids were first normalized by internal standards. The positively identified lipids were matched to one of 15 deuterated internal standards of different lipid classes that were spiked into samples before extraction, according to lipid class similarity and the expected retention time range for each class. Intensity ratios, i.e., the intensity of each lipid/feature divided by the intensity of the matched internal standard, were calculated for data normalization. Second, the identified lipids were median-normalized, i.e., the intensity ratio of each feature was divided by the median intensity ratio of all identified lipids within each sample experiment. Non-informative features (e.g., internal standards and common contaminants) and near-constant features were filtered out during data processing.

The following steps of data analysis were performed using R (version 4.3.2). Features filtered out included those with a relative standard deviation (RSD) greater than 25% for QC injections and features with near-constant values between groups, i.e., the top 30% of features with the lowest RSD among all samples. EigenMS normalization [38] was applied after several attempts of using other data transformation/normalization methods, such as log transformation, autoscaling, probabilistic quotient normalization, and variance stabilization normalization. However, only EigenMS normalization was robust enough to allow the detection of significantly different features between the comparison groups (LGA versus AGA) upon statistical analysis of the data. Each feature was tested for normality (from each comparison group) and for homogeneity of variance, using the Shapiro–Wilk test and Bartlett’s test, respectively. A *p* value > 0.01 was used to conclude a normal distribution and equal variances. Based on the results of these tests for each feature, univariate analysis was applied; with parametric (*t*-test) or non-parametric (Wilcoxon Rank Sum) tests and with either equal or non-equal variances. This was followed by False Discovery Rate (FDR) correction, using Benjamini–Hochberg adjustment. An adjusted *p* value < 0.05 was considered statistically significant. Insulin and glucose were also assessed similarly using this univariate analysis approach.

For differentially abundant (DA) features, i.e., significantly different between LGA and AGA, analysis of covariance (ANCOVA) was used to adjust for the effects of covariates: maternal BMI and age, fetal sex, and amniocentesis week. Linear regression models were fitted for each DA feature as the dependent variable and birth weight group (LGA/AGA) as the classification variable along with the covariates. To examine linear correlations of glucose and insulin with birth weight and DA features, Pearson and Spearman’s rank correlation testing were used for normally and non-normally distributed features, respectively. DA lipid features with identical identification were averaged for correlation analysis. Correction for multiple comparisons was also performed using Benjamini–Hochberg adjustment for ANCOVA and correlation testing. Log2 fold change was calculated for DA features as log2(mean feature intensity LGA/AGA). Principal component analysis (PCA) was applied to visualize the clustering of samples. Partial least squares discriminant analysis (PLS-DA) and variable importance in projection (VIP) were applied to extract features with high contribution to group separation. Only for PLS-DA analysis, the dataset was pareto-scaled, i.e., each feature was mean-centered and divided by the square root of its standard deviation. MixOmics (v6.25.1) and RVAideMemoire (v0.9-83-7) R packages, which offer sets of functions designed for multivariate analysis of biological data, were used to optimize the PLS-DA model and test its significance.

## 3. Results

### 3.1. Characteristics of Sample Population

Maternal and infant characteristics are shown in Table 1. Except for birth weight, no significant differences in maternal and infant characteristics were observed between LGA and AGA groups, mostly because of the matching criteria we applied.

### 3.2. Global Assessment of Lipidomic Features

Mass accuracy for data acquisition was assessed using internal standards. Out of 15 deuterated lipids/internal standards spiked in each sample and QC, 14 were detected in positive ionization and 12 were detected in negative ionization. The maximum mass error of internal standards detected was 1.44 ppm or 0.78 mDa for positive ionization and 0.81 ppm or 0.72 mDa for negative ionization, showing good mass accuracy. The average RSD (%) values for internal standards’ peak intensities as detected in QC samples were 13% in positive mode and 15% in negative mode. The average RSD (%) values of all detected features in QC samples before and after internal standard and median intensity normalization were 21.8% and 12%, respectively.

The analytical approach detected 4985 unique peaks in all samples, of which 2584 peaks were identified. In Tier 1 and Tier 2, 966 and 122 features were identified, respectively, at the species or molecular species level, with either full composition of fatty acyl/alkyl residues or summed composition if individual residues werenot specified in the source database. The remaining 1496 features were putatively identified in Tier 3 based on mass match and at the species level, i.e., only summed composition of fatty acyl/alkyl residues is provided. Multiple peaks were often annotated as the same lipid at the molecular species level (most identifications for Tiers 1 and 2) or at the species level (Tier 3), corresponding to similar lipids with minor differences in their structures, such as the position of double bonds, position of functional groups, and stereochemistry. Such differences between lipid isomers cannot be distinguished by the employed untargeted LC-MS/MS approach and require sophisticated targeted methods. Hence, several peaks had identical identifications.

**Figure 1:**
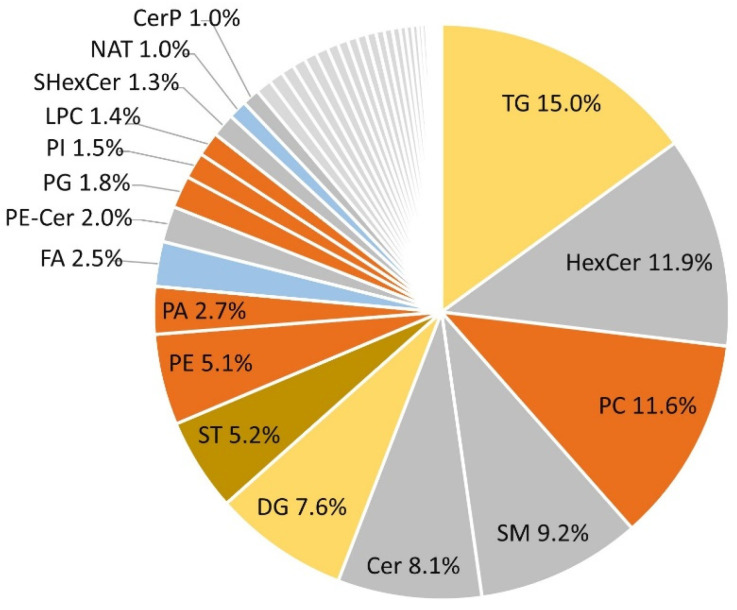
Relative abundance of the identified lipids’ subclasses. The abundance of each lipid class, among detected and identified peaks, is illustrated. Percentages were calculated by dividing the total count of identified lipids within each subclass by the total count of detected and identified peaks, i.e., 2584 peaks. Only subclasses with a relative abundance of ≥1% are shown. Abbreviations: Cer: ceramides, CerP: ceramide 1-phosphates, DG: diacylglycerols, FA: fatty acids, HexCer: hexosyl ceramides, LPC: lysophosphatidylcholines, NAT: N-acyl amines, PA: phosphatidic acids, PC: phosphatidylcholines, PE: phosphatidylethanolamines, PE-Cer: ceramide phosphoethanolamines, PG: phosphatidylglycerols, PI: phosphatidylinositols, SHexCer: sulfoglycosphingolipids, SM: sphingomyelins, ST: sterols, TG: triglycerides.

The identified lipids included 49 distinct lipid subclasses, of which triglycerides (TGs), hexosyl-ceramides, and phosphatidylcholines had the highest relative abundances, with percentages of 15%, 11.9%, and 11.5%, respectively (Figure 1). Sphingolipids and glycerophospholipids constituted more than 60% of all lipids identified. The distribution and total count of identified lipids within lipid categories and subclasses, along with their identification tiers, are provided in Supplementary data (Table S1, Figure S2). After applying the RSD filters, i.e., removing features with near-constant values and those with RSD > 25% in QC injections, 1780 features remained.

### 3.3. Univariate Analyses

**Figure 2:**
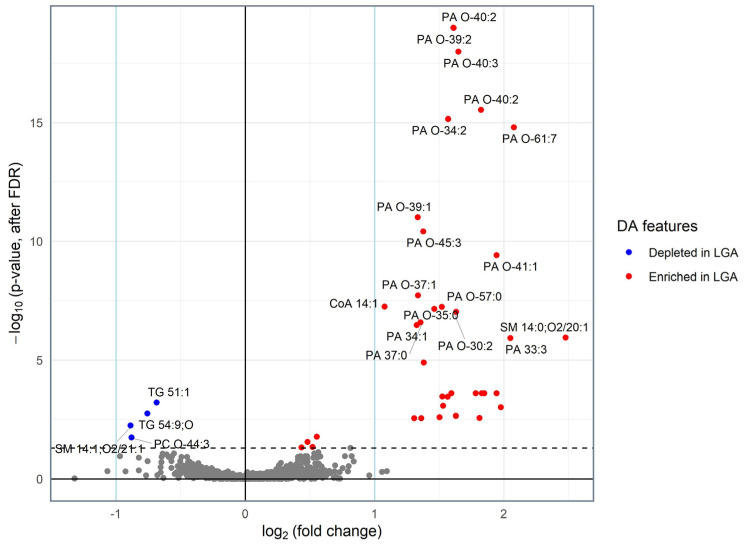
Volcano plot analysis of identified lipids. Features with an adjusted *p* value < 0.05 are colored in red if their intensities were higher in LGA compared to AGA AF and in blue if they were lower. Only features with an adjusted *p* value < 0.00001 are annotated. Abbreviations: CoA: acyl coenzyme A, PA: phosphatidic acid, SM: sphingomyelin.

Univariate analysis detected 42 features as significantly different between LGA and AGA samples. These features corresponded to 38 identified lipids, given that some features had similar identification (Table 2). All DA features were Tier 3-identified except for two features, which were Tier 1-identified as sphingomyelins (SMs). The majority of DA features (34 out of 42) were phosphatidic acid (PA) species, and they were all enriched in LGA AF samples. In addition, a few other species, including SM 14:0;O2/20:1, SM 39:1;O2, acyl-CoA (CoA 14:1), and wax ester (WE 22:1;O4), were elevated, while two TGs (TG 51:1 and TG 54:9;O), phosphatidylcholine (PC O-44:3), and SM 14:1;O2/21:1 were depleted in LGA samples compared to AGA. All DA features were still significantly different between LGA and AGA groups after adjusting for covariates using ANCOVA. A volcano plot was generated to visualize features’ intensity fold changes and statistical significance (Figure 2). Among DA features, SM 14:0;O2/20:1, followed by PA species and CoA 14:1 had the highest intensity fold changes (log2 fold change > 1). Insulin and glucose levels were not significantly different between LGA and AGA AF.

### 3.4. Multivariate Analyses

**Figure 3:**
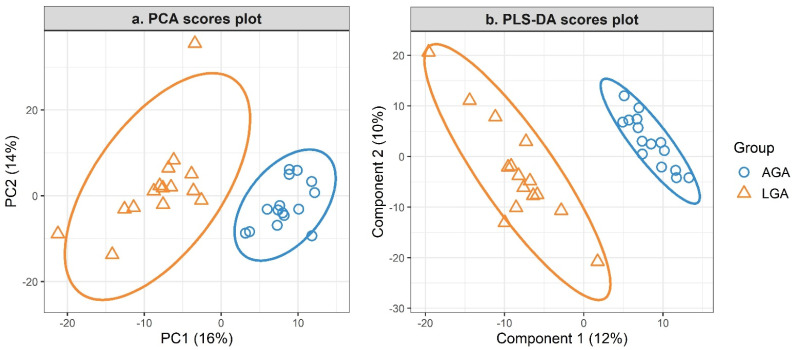
PCA and PLS-DA scores plots. (**a**) PCA 2D scores plot. (**b**) PLS-DA scores plot (mean classification error rate: 0.4 ± 0.12% based on an 8-component model, *p* value < 0.001 from 999 permutations based on 3-fold cross validation with 100 repeats).

**Figure 4:**
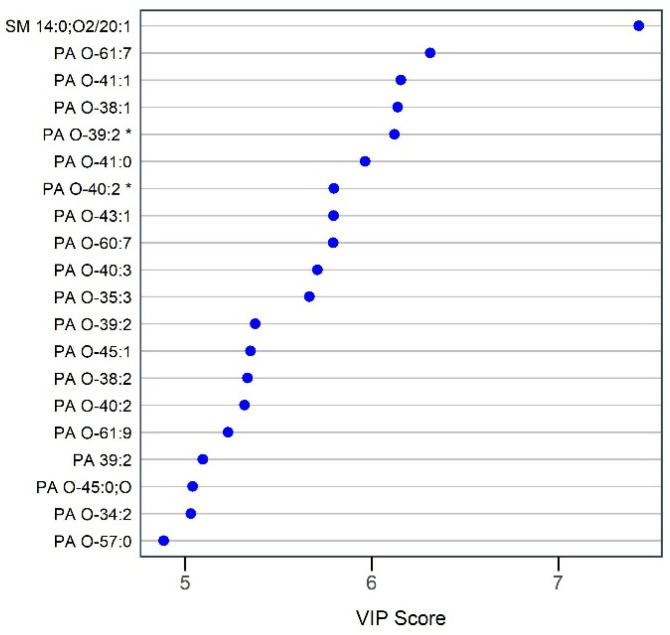
Variable importance in projection scores. The top 20 VIP scores of the first component from PLS-DA analysis are presented. All lipids displayed were significantly enriched in LGA compared to AGA AF. * Indicates features with replicate identification.

Unsupervised clustering of samples using PCA showed a clear separation between LGA and AGA groups as shown in PCA scores plot (Figure 3a), indicating different lipidomic trends between the two groups. A PLS-DA model was fit with 8 components (Figure 3b). The first two components explained 12% and 10% of the data variation. Three-fold cross validation with 100 repeats yielded a mean classification error rate of 0.4 ± 0.12%, and the model was significant upon permutation testing (*p* value = 0.001). VIP analysis showed that SM 14:0;O2/20:1 had the highest VIP score, followed by all DA PA species (Figure 4). All DA features had a VIP score >2. In other words, DA features had the highest contribution to group separation based on PLS-DA classification model. Results from correlation analysis showed that insulin and glucose did not correlate significantly with any of the DA features nor with birth weight (adjusted *p* value > 0.05).

## 4. Discussion

To our knowledge, this is the first study to profile the AF lipidome in relation to LGA as a pregnancy outcome. Untargeted lipidomics of AF revealed a unique signature of lipid metabolism characterizing fetal overgrowth during early pregnancy in healthy normal-weight mothers. This signature manifested mainly as a marked elevation of SM 14:0;O2/20:1, and many PA species with ether/ester bonds, different chain lengths, and degrees of saturation. This result is clearly different from the lipidomic features of maternal blood reported in association with higher birth weights. The general observation from previous studies indicates a reduction of maternal blood phosphatidylcholines and lysophosphatidylcholines in association with LGA birth weights [12,13,14,39]. Different prevailing features of LGA lipidome between maternal blood and AF are expected, given that AF uniquely reflects the interaction between maternal, placental, and fetal lipid metabolism, especially at early pregnancy.

### 4.1. Marked Elevation in Phosphatidic Acid Species

PA is a key intermediate in lipid metabolism and is implicated in insulin signaling pathways [40]. Cellular accumulation of PA was found to inhibit insulin signaling [41,42], an effect that seems to depend on PA composition of fatty acids and the synthetic pathways producing PA [43]. However, our sample of pregnant women were all healthy, with normal BMI, and none had any complications during pregnancy, like gestational diabetes. Therefore, it is not reasonable to assume that the observed increase in PA directly indicates maternal insulin resistance, although we cannot exclude the possibility of some degree of insulin resistance in these women. Also, elevated PA levels did not resemble a major feature of maternal blood or AF lipidome in association with gestational diabetes, which serves as a clear model for insulin resistance [20,29], although a few species have been reported to be elevated in patients with gestational diabetes [44].

A potential explanation of the observed elevation of PA in LGA AF could be related to enhanced placental stimulation of PA synthesis, secondary to increased maternal flux of TGs, non-esterified fatty acids (NEFAs), glucose, and growth factors such as insulin, which are positively associated with birth weight [45,46,47]. In that regard, both oleic acid [48] and insulin [49], acting as a growth factor, induce PA synthesis in primary human trophoblast cells (PHTs), which activates mammalian target of rapamycin (mTOR) [50]. Interestingly, activation of mTOR signaling was evident in the placentas of LGA infants [51]. Placental mTOR acts as a nutrient sensor, regulating nutrient delivery and fetal growth [48]. While amino acid uptake and transport are induced by mTOR activation in PHTs, the effect on placental lipid transport and metabolism is unknown [52]. It is noteworthy, however, that in cultured preadipocytes, activation of mTOR complex 1 promotes lipogenesis and inhibits lipolysis [53].

### 4.2. Alterations in Sphingomyelin Species

Among the DA features, two SMs were Tier 1-identified, i.e., with high confidence in structural identification. SM 14:0;O2/20:1 was markedly higher in LGA AF, with the highest VIP score and intensity fold change, while SM 14:1;O2/21:1 was lower. Not much is known about these two specific SM molecular species, and 14-carbon sphingoid bases are not common in human tissues [54]. However, the presence of SMs with 14-carbon sphingoid bases in AF can be well justified, given the high expression levels of serine palmitoyl transferase subunit 3 (SPTLC3) in placenta and trophoblast cells [55]. SPTLC3 has a high activity towards lauryl-CoA and myristoyl-CoA and primarily generates sphingoid bases with 14- or 16-carbon backbones [56]. It seems that monounsaturated SM species, at the sum composition, were particularly higher in LGA AF, as SM 14:0;O2/20:1 and SM 39:1;O2 were significantly elevated. In maternal blood, the opposite trend can be observed, as lower levels of monounsaturated SMs were reported in relation to birth weight among early pregnant mothers [13,57]. These opposite trends of alterations may indicate the divergence between the intrauterine and maternal lipid metabolism, which is likely the result of placental metabolism.

### 4.3. Alterations in Triglyceride and Fatty Acid Species

Another example of the unique intrauterine lipid profile that differs from the maternal side is the reduction of AF TGs. While maternal hypertriglyceridemia is frequently reported as a significant marker for macrosomia [45,58,59], our results showed that LGA infants had lower AF levels of two TG species, TG 54:9;O and TG 51:1. In line with this reduction is the negative association reported for cord blood TGs and birth weight [11,60]. Enhanced placental and fetal uptake and storage of fatty acids could explain the reduction of TGs observed in AF and cord blood. Furthermore, circulating myristic acid (C14:0), as a NEFA, was previously reported to correlate positively with maternal levels of TGs [61], which, as mentioned above, strongly predicts the risk of LGA. Myristic acid is known to activate several enzymes, such as desaturases, through protein myristoylation [62], which may explain the increase in its activated and monounsaturated form, acyl-CoA 14:1, we noted in LGA AF.

### 4.4. No Differences in Glucose and Insulin

Our results did not indicate any difference in AF concentration of glucose or insulin between LGA and AGA pregnancies, which is in line with previous studies [63,64,65]. However, one study reported that AF insulin is significantly associated with the risk of macrosomia among women with a positive glucose challenge test [66]. Furthermore, our analysis did not reveal any correlations between the DA lipids and AF concentration of glucose or insulin. Therefore, no evidence for glucose or insulin alterations in association with LGA pregnancies can be found based on the analysis of our AF samples.

### 4.5. Limitations

Among the limitations of this study is the small sample size, which aligns with the exploratory nature of the work. The lack of information on the dietary intake of participants and their glycemic and lipid profiles has limited our ability to draw stronger conclusions of LGA AF lipidome in relation to maternal metabolic health. The advanced maternal age of participants may limit the generalizability of our findings. Long storage duration of our AF samples is another limitation. However, given that all samples were stored for similar durations, it can be assumed that the effects of storage on the lipidome were consistent across all samples, hence, maintaining the relationships with birth weight. Aliquoting AF samples prior to storage at −80 °C also helped minimize the freeze–thaw cycles. Furthermore, although the employed identification method used a nine-tier approach to filter and select the best identification choice for each detected feature, most features identified as DA, such as PA species, were Tier-3 identified. This identification tier was based on mass match rather than MS/MS identification, which implies lower confidence in the identification. However, the fact that 34 DA features were consistently identified as PA reduces the possibility of misidentifying PA as an important DA lipid.

## 5. Conclusions

While insulin and glucose were not different between LGA and AGA AF, several lipid species were DA, of which PA species were dominantly and markedly elevated in LGA AF. We suspect that elevated levels of maternal lipids and growth factors, such as insulin, induce higher placental synthesis of PA, leading to the observed elevation of PA in LGA AF. Furthermore, elevated SM 14:0;O2/20:1 strongly distinguished LGA from AGA AF; however, the role and the significance of this SM need further investigation. Overall, our results may provide a mechanistic clue about the positive association between birth weight and maternal circulating lipids and growth factors. Furthermore, this study signifies the role of altered lipid metabolism in promoting augmented fetal adiposity. Future research with both comprehensive and targeted lipidomic profiling is needed to establish our results and expand upon them. More focus on the early stages of pregnancy, tissues from the intrauterine compartment, and the metabolic pathways involved in the synthesis of PA and SMs with 14-carbon sphingoid bases may provide further insights into the underlying mechanisms promoting macrosomia.

## Figures and Tables

**Table 1:** Maternal and infant characteristics.

	LGA (n = 15)	AGA (n = 15)
**Maternal**		
Age (years)	37.2 ± 1.5 (35.2–40.0)	37.3 ± 1.7 (35.0–40.6)
Pre-pregnancy weight (kg)	61.6 ± 7.4 (48.2–80.0)	58.5 ± 6.6 (47.6–70.3)
Height (m^2^)	1.68 ± 0.08 (1.58–1.94)	1.64 ± 0.09 (1.51–1.80)
BMI (kg/m^2^)	21.7 ± 1.7 (18.1–24.7)	21.7 ± 1.6 (18.5–24.0)
Gestational age at amniocenteses (wk)	15.2 ± 0.8 (14.0–17.0)	15.2 ± 0.7 (14.0–16.5)
AF glucose (mmol/L)	4.3 ± 2.0 [n = 13] (2.3–8.1)	4.09 ± 1.05 (2.1–6.1)
AF insulin (pmol/L)	3.7 ± 3.2 [n = 13] (0.3–10.2)	2.84 ± 2.83 (0.3–9.5)
**Infant**		
Sex (n = female/male)	5/10	5/10
Birth weight (grams) *	4314 ± 333 (3930–5310)	3532 ± 346 (3134–4020)
Gestational age at birth (wk)	40.2 ± 1.1 (38.5–41.9)	39.74 ± 0.7 (38.1–40.8)

Data are presented as mean ± SD, followed by (minimum–maximum). * Indicates a significant difference between LGA and AGA (*p* value < 0.05).

**Table 2:** Differentially abundant features between LGA and AGA infants.

Feat. No	Identified Feature/Lipid	Log2 Fold Change	Adjusted *p*-Value
Features enriched in LGA AF			
1	PA O-39:2 ^R^	1.63	<0.001
2	PA O-40:2 ^R^	1.64	<0.001
3	PA O-40:3 ^R^	1.65	<0.001
4	PA O-40:2 ^R^	1.81	<0.001
5	PA O-34:2	1.59	<0.001
6	PA O-61:7	2.11	<0.001
7	PA O-39:1	1.32	<0.001
8	PA O-45:3	1.37	<0.001
9	PA O-41:1	1.96	<0.001
10	PA O-37:1	1.37	<0.001
11	CoA 14:1	1.08	<0.001
12	PA O-57:0	1.51	<0.001
13	PA O-35:0	1.45	<0.001
14	PA O-30:2	1.61	<0.001
15	PA 37:0	1.37	<0.001
16	PA 34:1	1.35	<0.001
17	SM 14:0;O2/20:1	2.51	<0.001
18	PA 33:3	2.08	<0.001
19	PA O-45:2	1.38	<0.001
20	PA 39:2	1.83	<0.001
21	PA O-38:2	1.61	<0.001
22	PA O-39:2 ^R^	1.83	<0.001
23	PA O-41:0	1.93	<0.001
24	PA O-43:1	1.79	<0.001
25	PA O-45:1	1.55	<0.001
26	PA O-61:9	1.56	<0.001
27	PA O-45:0;O	1.51	<0.001
28	PA O-38:1	1.95	<0.001
29	PA O-39:0	1.66	0.002
30	PA O-60:7	1.60	0.002
31	PA O-36:0	1.52	0.003
32	PA O-35:3	1.84	0.003
33	PA O-40:3 ^R^	1.33	0.003
34	PA O-40:3 ^R^	1.35	0.003
35	WE 22:1;O4	0.54	0.017
36	PA O-62:12	0.47	0.028
37	SM 39:1;O2	0.51	0.045
38	PA 27:2	0.45	0.047
Features depleted in LGA AF			
39	TG 51:1	–0.68	<0.001
40	TG 54:9;O	–0.78	0.002
41	SM 14:1;O2/21:1	–0.88	0.006
42	PC O-44:3	–0.89	0.018

All features listed were Tier 3 identified, except SM 14:0;O2/20:1 and SM 14:1;O2/21:1 which were Tier 1 identified, i.e., based on MS/MS identification. ^R^ Indicates features with replicate identification. Abbreviations: PA: phosphatidic acid, CoA: acyl coenzyme A, SM: sphingomyelin, WE: wax ester, TG: triglycerides, PC: phosphatidylcholine. Shorthand notation of lipid species; PA 37:0 denotes a PA with a total of 37 carbons and 0 double bonds in its two ester-linked fatty acyl chains; PA O-39:2 denotes a PA with an ester-linked fatty acid group and an ether/vinyl ether-linked fatty alcohol group containing a total of 39 carbons and 2 double bonds; SM 14:0;O2/20:1 denotes a SM in which the sphingoid base contains 14 carbons, 0 double bonds, and 2 hydroxyl groups, and the fatty acid contains 20 carbons and 1 double bond; SM 39:1;O2 denotes a SM in which the sphingoid base and fatty acid contain a total of 39 carbons, 1 double bond, and 2 hydroxyl groups.

## Data Availability

The raw data supporting the conclusions of this article will be made available by the corresponding author on reasonable request.

## Supplementary Materials

The following supporting information can be downloaded at: https://www.mdpi.com/article/10.3390/nu16223804/s1, Figure S1: Flowchart of sample selection from AF biobank; Figure S2: Distribution of identified lipids among lipid subclasses and identification tiers; Table S1: Total count of identified lipids within lipid categories, subclasses, and identification tiers.
