# Supplementary material for: Untargeted Lipidomic Profiling of Amniotic Fluid Reveals Dysregulated Lipid Metabolism in Healthy Normal-Weight Mothers with Fetal Macrosomia
Source: Nutrients. 2024 Nov 6;16(22):3804. doi: 10.3390/nu16223804 (PMC11597394; doi:10.3390/nu16223804)
Supplement: Supplementary file 1 [file nutrients-16-03804-s001.zip › nutrients-3249134-supplementary.pdf]

Untargeted Lipidomic Profiling of Amniotic Fluid Reveals Dysregulated Lipid Metabolism in Healthy Normal-Weight Mothers with Fetal Macrosomia

Isra'a Haj-Husein *, Stan Kubow and Kristine G. Koski

Supplementary Data

- Methods: Sample selection

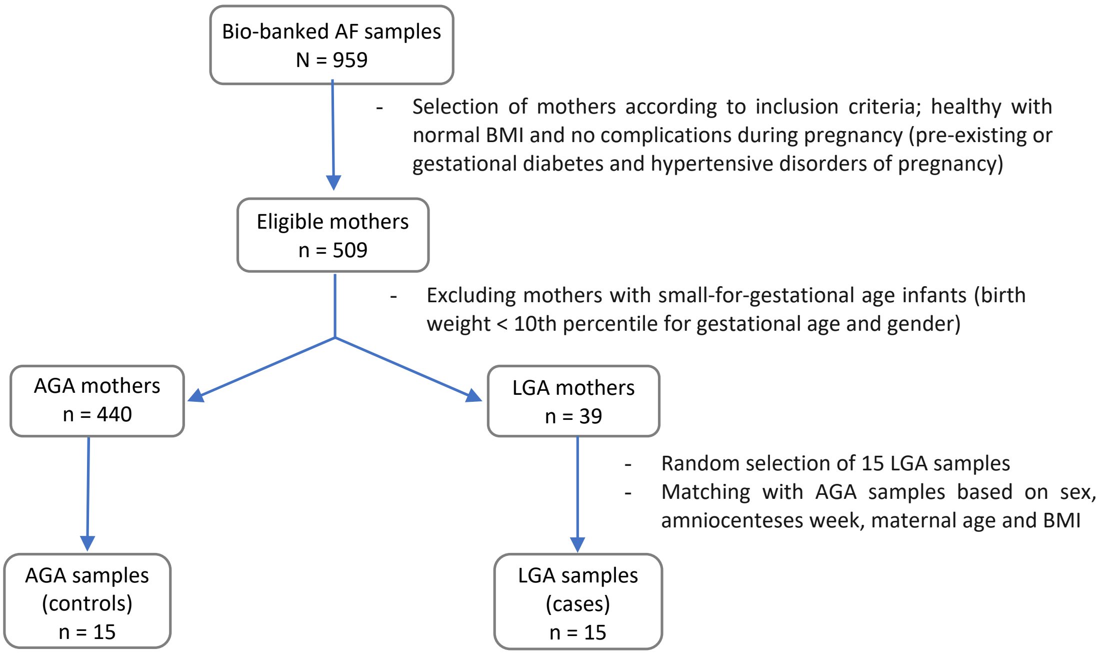

Figure S1: Flowchart of sample selection from AF biobank

- Results: Identified lipidomic features of AF

Table S1: Total count of identified lipids within lipid categories, subclasses, and identification tiers

Lipid Category	Subclass	Tier 1	Tier 2	Tier 3	Total count (%)
Sterols	ST	4	10	121	135
Sterols	CE			1	1
Sterols-Total					136 (5.3%)
Sphingolipids	ACer			11	11
Sphingolipids	Cer	79	2	128	209
Sphingolipids	CerP			25	25
Sphingolipids	GlcCer			4	4
Sphingolipids	HexCer	128	7	172	307
Sphingolipids	PE-Cer	7	4	41	52
Sphingolipids	PI-Cer			14	14
Sphingolipids	SHexCer	11	2	21	34
Sphingolipids	SPBP			1	1
Sphingolipids	SM	130	31	78	239
Sphingolipids	M(IP)2C			1	1
Sphingolipids	MIPC			2	2
Sphingolipids-Total					899 (34.8%)
Glycerophospholipids	LPA			3	3
Glycerophospholipids	LPC	22	7	6	35
Glycerophospholipids	LPE	7	10	2	19
Glycerophospholipids	LPG		1	5	6
Glycerophospholipids	LPI			6	6
Glycerophospholipids	PA	3	2	64	69
Glycerophospholipids	PC	237	1	62	300
Glycerophospholipids	PE	95	2	35	132
Glycerophospholipids	PG	3		44	47
Glycerophospholipids	PI	18	3	17	38
Glycerophospholipids	PIP			16	16
Glycerophospholipids	PS	4		10	14
Glycerophospholipids	BMP	1		1	2
Glycerophospholipids	GP		3		3
Glycerophospholipids-Total					690 (26.7%)
Glycerolipids	TG	133	1	254	388
Glycerolipids	DG	25	3	168	196
Glycerolipids	MG	1	1	7	9
Glycerolipids	GlcADG	12			12

Glycerolipids	DGCC		1		1
Glycerolipids	DGDG		1		1
Glycerolipids	MGDG	13	7	1	21
Glycerolipids	SQDG	1			1
Glycerolipids-Total					629 (24.3%)
Fatty Acyls	CAR	1	1	16	18
Fatty Acyls	CoA			1	1
Fatty Acyls	FA	18	6	41	65
Fatty Acyls	FAHFA	9	1	4	14
Fatty Acyls	HC			8	8
Fatty Acyls	NA	1	9	8	18
Fatty Acyls	NAE			12	12
Fatty Acyls	NAT			27	27
Fatty Acyls	FAG			1	1
Fatty Acyls	WE			15	15
Fatty Acyls-Total					179 (6.9%)
Other	PK			19	19
Other	PR		1	22	23
Other	SL	3	5	1	9
Other-Total					51 (2%)
Grand Total		966	122	1496	2584

Percentage of relative abundance for each lipid class was calculated based on the total number of identified features (n = 2584). Abbreviations: ACer: Acylceramides, BMP: Monoacylglycerophosphomonoradylglycerols, CAR: Fatty acyl carnitines, CE: Steryl esters, Cer: Ceramides, CerP: Ceramide 1-phosphates, CoA: Fatty acyl CoAs, DG: Diglycerides, DGCC: Hydroxymethyl-choline, DGDG: Digalactosyldiacylglycerols, FA: Fatty acids, FAG: Fatty acyl glycosides of mono- and disaccharides, FAHFA: Fatty acid estolides, GlcADG: Glycosyldiacylglycerols, GlcCer: Glucuronosphingolipids, GP: Other Glycerophospholipids, HC: Hydrocarbons, HexCer: Hexosyl ceramides, LPA: Lysophosphatidic acids, LPC: Lysophosphatidylcholines, LPE: Lysophosphatidylethanolamines, LPG: Lysophosphatidylglycerols, LPI: Lysophosphatidylinositols, MG: Monoglycerides, MGDG: Monogalactosyldiacylglycerols, MIPC: Ceramide phosphoinositols, M(IP)2C: Ceramide phosphoinositols, NA: Nitrogenated fatty acids, NAE: N-acyl ethanolamines, NAT: N-acyl amines, PA: Phosphatidic acids, PC: Phosphatidylcholines, PE: Phosphatidylethanolamines, PE-Cer: Ceramide phosphoethanolamines, PG: Phosphatidylglycerols, PI: Phosphatidylinositols, PI-Cer: Ceramide phosphoinositols, PIP: Glycerophosphoinositol monophosphates, PK: Polyketides, PR: Prenol Lipids, PS: Phosphatidylserines, SHexCer: Sulfoglycosphingolipids, SL: Saccharolipids, SM: Sphingomyelins, SPBP: Sphingoid base-1 phosphates, SQDG: Sulfoquinovosyldiacylglycerols, ST: Sterol, TG: Triglycerides, WE: Wax esters and diesters.

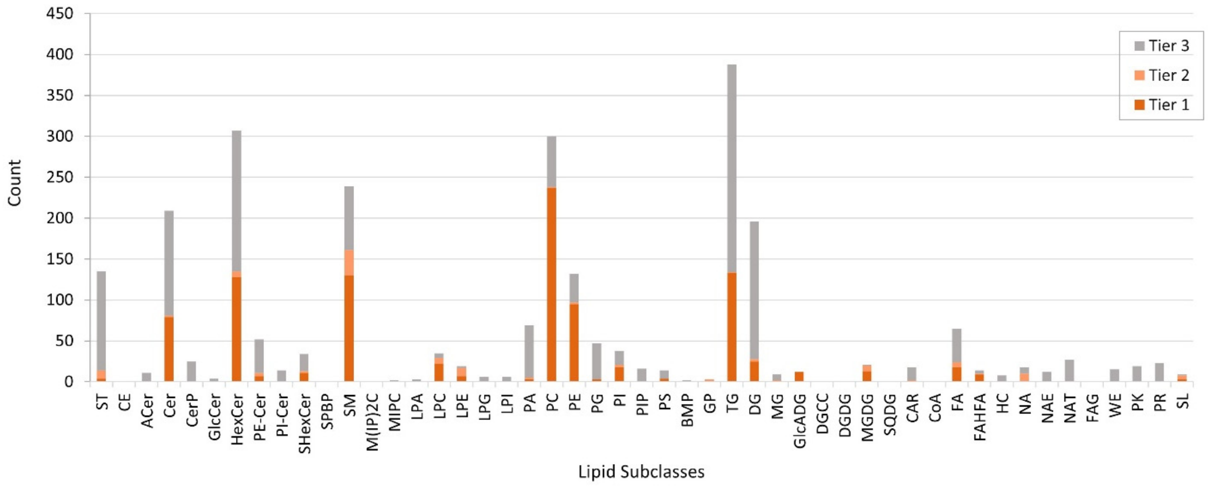

Figure S2: Distribution of identified lipids among lipid subclasses and identification tiers
